# Observations on Some Pharmaceutical Preparations of Opium

**Published:** 1820-04

**Authors:** 


FOR THE LONDON MEDICAL AND PHYSICAL JOURNAL.

### *Observations on some Pharmaceutical Preparations of Opium.*

*By* A. B. G.

IN some recent pharmacological publications,* two preparations of opium, not admitted in the English Pharmacopœias, have been divulged to the profession, with some degree of complacency at this fresh instance of modern acuteness in unveiling the most hidden mysteries of empiricism. I allude to the long-celebrated "black drop," and to the "liquor opii sedativus," yet young in fame. The composition of both these medicines having thus ceased to be a secret, I naturally became desirous of knowing how far their reputed inventors had a claim to that distinction; and for this purpose I turned to my old Latin books, an inexhaustible source of remedies for quacks and regulars, where, after a short search, I found the first of those preparations in Vanhelmont, under the name of "laudanum cydoniatum;" and the second in Etmuller, qualified by the appellation of "magisterium opii."

That the public may judge of the accuracy of this statement, I shall beg leave to subjoin the formula for Vanhelmont's preparation, as well as that for the "black drop," attributed to Mr. Tonstall, of Bishop Auckland, according to Dr. Armstrong,† who first gave publicity to it. ℞. Opii pulveris ℥ iv. succi cydoniorum recent. lb. iv.; digerantur simul per tres septimanas; tum adde caryoph. arom. nucis mosch. *aa* ℥ i.; ebulliantur, et digerantur rursus per septimanam unam, et initio ultioris diei adde croci ℥ i.; sacchari ℥ iv.; tunc filtra et in loco calido evaporetur ad tertiam partem. The formula for the black drop is this: "Take half a pound of opium sliced; three pints of good verjuice; one and a half ounce of nutmegs; half an ounce of saffron; boil them to proper thickness; then add a quarter of a pound of sugar, *and two spoonfuls of yeast.* Set the whole in a warm place near the fire for six or eight weeks, then place it in the open air until it becomes a syrup, &c. In the editions of Vanhelmont to which I have had access, I have not been able to find the *fermentum* (yeast) of the latter preparation; but I strongly suspect that Mr. Tonstall took his medicine

* *Thomson's Dispensatory,* edit. 2, p. 292. *Dr. Paris's Pharmacologia,* edit. 2, p. 313.

† *Dr. Armstrong's Practical Illustrations of Typhus.*

from the treatise on Chemistry by Wilson, where the author had most probably added the yeast to promote fermentation; as I find that Jones, in his book on opium,* condemns this addition of Wilson as improper and unnecessary; since sugar, a vegetable acid, and heat, as directed in Vanhelmont's original formula, are quite sufficient to produce that phenomenon.

One drop of the "black drop" is said to be equal to four drops of laudanum, or, in other words, five drops to equal one grain of opium; and it is a curious circumstance, that, in an interleaved copy of Jackson's Enchiridion Medicum (1705), which there is every reason to believe to have been the author's own copy, an observation to the same purport is found in one of the many manuscript additions with which that copy is enriched. After inspecting Vanhelmont's Latin formula for the *laudanum cydoniatum*, the author remarks, "Guttæ decem hujus tincturæ, possunt gr. i. laudan. thebaic. (*i.e.* pure opium) æquipollere." This copy of Jackson's Enchiridion belonged to the late eminent physician Sir Walter Farquhar, and came into my possession, with many more of his curious medical books, as a present from Sir Thomas Farquhar.

With regard to the "liquor opii sedativus," there is every reason to believe, as I have before stated, that the *magisterium opii*† of the Pharmacopœia Danielis Ludovici, commented upon by Etmuller, in his works, vol. i. p. 997, is a similar, if not altogether an identical, preparation, consisting chiefly in submitting opium to the action of vinegar. Etmuller enumerates the properties of this preparation thus: "Remedium est temperantivum et frigens et quasi liquor quidam terræ foliatæ tartari sociatus opio, hincque diaphoreticus, temperans, et simul anodynus."

If the medical men of the present age condescended to study the language which seems the most adapted for their profession, and thus secured to themselves a ready access to the oldest repositories of medical learning, they might, as occasion required, bring again into use valuable medicines, which, being now lost to the generality of the practitioners unacquainted with Latin, are from time to time revived by quacks only, or by some aspiring apothecary, with an affectation of novelty.

15*th February*, 1820.

---

* *Mysteries of Opium revealed, &c.* by Jones. Lond. 1700.

† Magisterium opii fit solvendo opium in aceto et præcipitando cum sale tartari.

